# Assessing the Effects of Participant Preference and Demographics in the Usage of Web-based Survey Questionnaires by Women Attending Screening Mammography in British Columbia

**DOI:** 10.2196/jmir.5068

**Published:** 2016-03-22

**Authors:** Rebecca Mlikotic, Brent Parker, Rasika Rajapakshe

**Affiliations:** ^1^ British Columbia Cancer Agency Sindi Ahluwalia Hawkins Centre for the Southern Interior Kelowna, BC Canada; ^2^ Interior Health Authority Department of Surgical Services Kelowna, BC Canada; ^3^ Faculty of Medicine Department of Surgery University of British Columbia Vancouver, BC Canada; ^4^ Irving K. Barber School of Arts and Sciences Department of Computer Science (Unit 5) University of British Columbia, Okanagan Campus Kelowna, BC Canada

**Keywords:** patient preference, patient reported outcomes, patient reported outcome measures, questionnaires, Internet, Web-based system, survey methods, breast cancer screening

## Abstract

**Background:**

Increased usage of Internet applications has allowed for the collection of patient reported outcomes (PROs) and other health data through Web-based communication and questionnaires. While these Web platforms allow for increased speed and scope of communication delivery, there are certain limitations associated with this technology, as survey mode preferences vary across demographic groups.

**Objective:**

To investigate the impact of demographic factors and participant preferences on the use of a Web-based questionnaire in comparison with more traditional methods (mail and phone) for women participating in screening mammography in British Columbia, Canada.

**Methods:**

A sample of women attending the Screening Mammography Program of British Columbia (SMPBC) participated in a breast cancer risk assessment project. The study questionnaire was administered through one of three modes (ie, telephone, mail, or website platform). Survey mode preferences and actual methods of response were analyzed for participants recruited from Victoria General Hospital. Both univariate and multivariate analyses were used to investigate the association of demographic factors (ie, age, education level, and ethnicity) with certain survey response types.

**Results:**

A total of 1192 women successfully completed the study questionnaire at Victoria General Hospital. Mail was stated as the most preferred survey mode (509/1192, 42.70%), followed by website platform (422/1192, 35.40%), and telephone (147/1192, 12.33%). Over 80% (955/1192) of participants completed the questionnaire in the mode previously specified as their most preferred; mail was the most common method of response (688/1192, 57.72%). Mail was also the most preferred type of questionnaire response method when participants responded in a mode other than their original preference. The average age of participants who responded via the Web-based platform (age 52.9, 95% confidence interval [CI] 52.1-53.7) was significantly lower than those who used mail and telephone methods (age 55.9, 95% CI 55.2-56.5; *P*<.001); each decade of increased age was associated with a 0.97-fold decrease in the odds of using the website platform (*P*<.001). Web-based participation was more likely for those who completed higher levels of education; each interval increase leading to a 1.83 increase in the odds of website platform usage (*P*<.001). Ethnicity was not shown to play a role in participant preference for the website platform (*P*=.96).

**Conclusions:**

It is beneficial to consider participant survey mode preference when planning to collect PROs and other patient health data. Younger participants and those of higher education level were more likely to use the website platform questionnaire; Web-based participation failed to vary across ethnic group. Because mail questionnaires were still the most preferred survey mode, it will be important to employ strategies, such as user-friendly design and Web-based support, to ensure that the patient feedback being collected is representative of the population being served.

##  Introduction

Survey questionnaires are one of the most prominent data collection methods in cancer health and epidemiological research. Not only do they provide a means for evaluating patient care and treatment efficacy, but they also allow for valuable communication between physicians and patients [1]. However, the collection of patient reported outcomes (PROs) and other clinical data can be both difficult and costly, resulting in the need for more sustainable collection means [2]. Current typical survey methods include in-person, phone, mail, and Internet applications. These methods are used both exclusively (single-mode) and in combination (mix-mode); preference is generally shown for mix-mode questionnaires as they typically lead to higher response rates and reduced costs [3-5].

Increased use of Internet applications within society and health care has led to a shift toward Web-based surveying methods from more traditional methods such as mail or telephone. These methods include Web-based questionnaires, which can be predesigned as a HyperText Markup Language (HTML) form or assembled from a question database, in addition to mobile questionnaires that operate through a tablet or smart phone [6-8]. Web-based methods have also been used in combination with more traditional methods, such as in computer-assisted telephone interviewing (CATI) [9]. Overall, this shift is due to both the speed and ease of Web-based questionnaire delivery [4,10-14]. Web-based platforms also allow for potentially larger sample sizes to be reached [11,12,15], and a more economical dissemination of the questionnaire compared with more traditional methods [4,10-13]. Survey questions can also be dynamically adjusted and their allowable answers varied, based on the answers given to previous questions. These methods also allow for automatic upload of patient responses into databases, reducing human error associated with data entry, and thus increasing data quality [12,13]. Occurrences of missing data are also minimized, as certain applications prevent patients from proceeding until the previous survey question has been completed [1].

However, Web-based questionnaire applications also present certain limitations. Meaningful patient consent is often difficult to ensure, in addition to the successful integration of PRO data into other medical information records [2]. While Web-based questionnaire delivery costs may appear more economical than other methods, they involve the cost of professional programmers and the maintenance of data security [2,5]. Nonresponse bias is also associated with use of Web-based technologies, as patient participation is often due to factors such as Internet access, in addition to a patient’s familiarity and comfort level in using Web platforms [2,12,13,16]. Furthermore, demographic groups differ in their personal preferences for and attitudes toward various survey modes. Developing an understanding of which demographic factors influence Web-based application participation will enable both clinicians and researchers to better predict how their platforms will be received in certain settings.

The Screening Mammography Program of BC (SMPBC) is the longest running organized breast screening program in Canada. It was initiated as a single fixed site in Vancouver in July, 1988 [17]. By 2010, geographic coverage of the province was achieved with 42 services employing a variety of delivery modes including four mobile vans. Asymptomatic female residents of BC, age 40 and older, are eligible to attend for regular, bilateral, two-view, screening mammograms in this publically funded program. The SMPBC database contains data on all invasive or in situ breast cancers diagnosed in women who have previously attended the SMPBC and conducts a central pathology review on those cancers. It also uses a paper questionnaire to collect Gail model risk factors from the attending women since its inception in 1988. However, it has been shown that the Tyrer-Cuzic (TC) model is more accurate than the Gail model in predicting breast cancer risk [18,19]. Therefore, a study was conducted during 2009 and 2010 to collect TC model risk factors from a sample of women attending the SMPBC. By doing so, the risk distribution for that population was estimated in order to assess the need for additional magnetic resonance images required to screen high-risk women [20]. The participants were given three options for participating in the study: mail-in paper questionnaire, Web-based questionnaire, or phone interview. Our current study attempts to better characterize the effect of survey mode preference and demographic factors that influenced the usage of a Web-based questionnaire in comparison with more traditional methods (phone and mail) for women participating in the original study. Because the current standard of practice of the SMPBC is to collect breast cancer risk factors using a paper-based questionnaire, it is important to understand these characteristics so that the feasibility of administering Web-based questionnaires in the future can be evaluated. This information may also be helpful to others involved in gathering breast cancer risk factors needed for personalizing breast screening strategies.

##  Methods

### Study Population and Recruitment

Approval of the project was gained through the British Columbia Cancer Agency Research Ethics Board (UBC BCCA REB Certificate #H09-00681). The study data was collected as part of a previous project, where a breast cancer risk assessment was conducted for the women participating in the SMPBC [20]. The focus of this manuscript is a subpopulation of women from the assessment who were attending screening mammography at Victoria General Hospital in Victoria, British Columbia (BC) from August 2009 to January 2010. All participants gave voluntary informed consent prior to study involvement, and were 40 to 79 years of age at the time of the study. The study involved the development and administration of a questionnaire to collect personal information related to common risk factors for breast cancer of women attending the SMPBC [20]. Within the consent form, participants were also asked to state their preferred method of response as either mail, telephone, or the study website platform, should they agree to participate [21]. The questionnaire was distributed by both clerks and on-site volunteers, with each participant invited to complete it at her own convenience using her preferred method of response. All participant information and responses were stored in a study database. Once the questionnaire was completed, an unique study identification was assigned to each participant to ensure confidentiality and anonymity.

### Statistical Analysis

In order to better understand patient preferences and factors influencing questionnaire response type, both univariate and multivariate analyses were used to explore demographic factors associated with certain response types. All analyses were performed using STATA 13.1.

#### Univariate Analysis

Survey mode preferences were compared with actual response type. Age was grouped into four cohorts: 40 to 49, 50 to 59, 60 to 69, and 70 to 79. The distribution of questionnaire response types was determined for three demographic factors: age, education level, and ethnicity. Mean participation rates for each demographic variable were analyzed using the Mann-Whitney U test, Wilcoxin Rank-Sum test, or Fisher’s exact test.

#### Multivariate Analysis

Of specific interest was improved characterization of the association between the available demographic factors and Internet participation, and differences between proportions were assessed using chi-square and Fisher’s exact tests. Multivariate logistic regression was used to evaluate the association between Web-based survey usage versus other modes, adjusting for age, education level, and ethnicity. Each of these three variables was forced into the logistic model. Of the demographic variables, interaction was found only between education and age. Statistical significance was defined as *P*<.05.

## Results

### Participant Survey Mode Preference

A total of 1192 women successfully completed the study questionnaire at Victoria General Hospital. Of the three survey modes, distribution by mail was the most preferred (509/1192, 42.70%), followed by website platform (422/1192, 35.40%), and telephone (147/1192, 12.33%; Table 1). Approximately 10% of participants (114/1192, 9.56%) did not specify a preferred method of response in their consent form.

**Figure 1:**
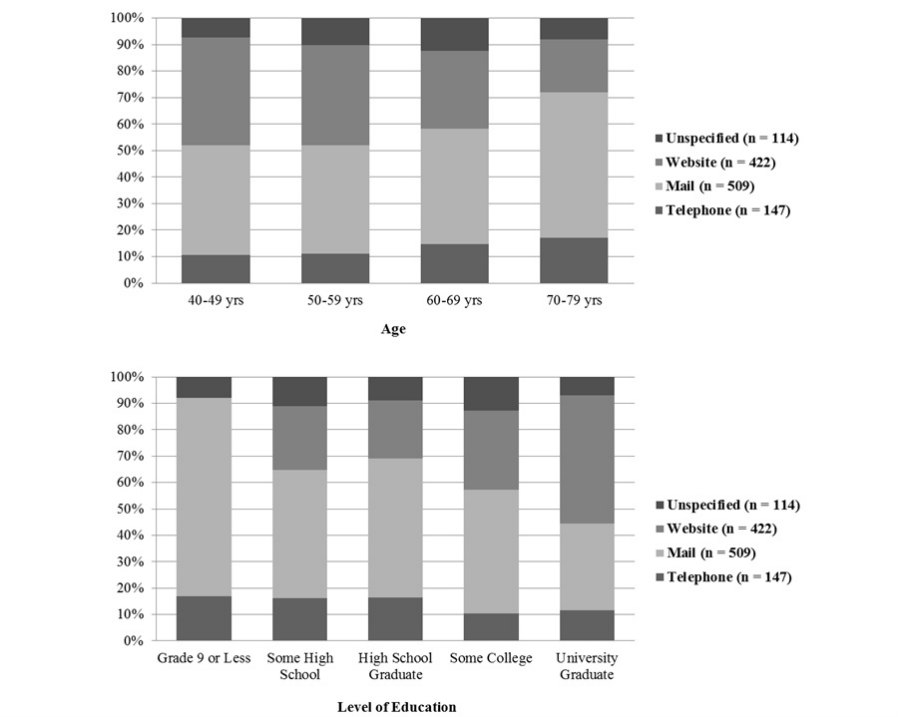
Percent distribution of participant demographics for each preferred method of questionnaire response (n = 1192).

Survey mode preference differed between younger (<60 years) and older (>60 years) patients, as preference for mail and telephone questionnaires strengthened with age (Figure 1). In contrast, the Web-based option became less preferred with each subsequent age grouping. Higher levels of post-secondary education led to a stronger preference for Web-based questionnaires, while the opposite trend was observed for mail. With regards to ethnicity, nearly all groups expressed a preference for the mail mode.

### Variation Between Preferred and Actual Participant Survey Response

**Figure 2:**
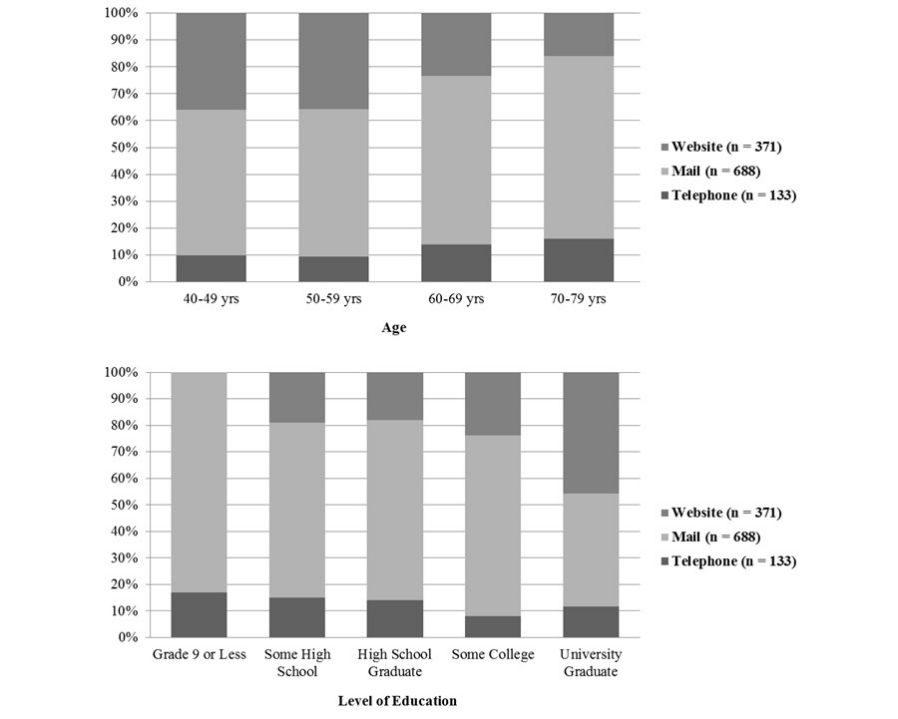
Percent distribution of participant demographics for each actual questionnaire response type (n = 1192).

In all, 80.12% (955/1192) of patients completed the questionnaire in the mode that was previously specified as their most preferred method (Tables 2, 3, Figure 2). For those who responded in a method other than their original preference, mail was the most common response type. In addition, for those who did not specify a preferred response method, mail was also the most common response type (Table 3).

**Table 1:** Preferred method of questionnaire response stratified by participant demographics.

		Telephone	Mail	Website	Unspecified	Total
		n=147	n=509	n=422	n=114	n=1192
Age						
	40-49	44	174	171	30	419
	50-59	41	149	139	37	366
	60-69	49	144	97	41	331
	70-79	13	42	15	6	76
Education						
	Grade 9 or Less	2	9	0	1	12
	Some high school	10	30	15	7	62
	High school diploma	37	119	50	20	226
	Some college	42	192	123	52	409
	University degree	56	159	234	34	483
Ethnicity						
	White^a^	133	478	386	111	1108
	Other	6	10	20	3	39
	East/Southeast Asian	4	10	8	0	22
	First Nation/Métis	2	5	5	0	12
	South Asian	1	4	2	0	7
	African/African American	1	2	1	0	4

^a^White = British/French/Irish/Scottish/Welsh/(Northern, Southern, Eastern, or Western) European.

**Table 2:** Actual method of questionnaire response stratified by participant demographics.

		Telephone	Mail	Website	Total
		n=133	n=688	n=371	n=1192
Age					
	40-49	41	227	151	419
	50-59	34	201	131	366
	60-69	46	208	77	331
	70-79	12	52	12	76
Education					
	Grade 9 or Less	2	10	0	12
	Some high school	9	41	12	62
	High school diploma	32	153	41	226
	Some college	33	279	97	409
	University degree	57	205	221	483
Ethnicity					
	White^a^	116	649	343	1108
	Other	7	13	19	39
	East/Southeast Asian	4	13	5	22
	First Nation/Métis	4	6	2	12
	South Asian	1	5	1	7
	African/African American	1	2	1	4

^a^White = British/French/Irish/Scottish/ Welsh/(Northern, Southern, Eastern, or Western) European.

**Table 3:** Comparison of participant-stated preferences and actual survey response types.

		Actual Response Type			
		Telephone	Mail	Website	Total
		n=133	n=688	n=371	n=1192
Preferred response type					
	Telephone	120	23	4	147
	Mail	5	492	12	509
	Website	8	71	343	422
	Unspecified	0	102	12	114

### Actual Patient Survey Response

The study uptake at the Victoria General Hospital for which this analysis is performed was found to be 47% [20]. This is comparable with the on-site study uptake of 31% to 50% in a similar risk assessment study conducted by Evans and colleagues [22]. Of the questionnaires that were completed, 57.72% (688/1192) were submitted via mail, followed by the 31.12% (371/1192) completed using the website platform, and the 11.16% (133/1192) by telephone (Tables 2, 3). The overall average age for participation was 55 years (95% confidence interval [CI] 54.4-55.5). The average age of participants was significantly lower for those who responded via the website platform (52.9, 95% CI 52.1-53.7) compared with those who used telephone or mail (55.9, 95% CI 55.2-56.5; *P*<.001).

Each additional level of education showed an increased number of respondents, with university graduates being the largest group to complete the questionnaire (483/1192, 40.52%). Web-based participation was also more likely in higher education groups (*P*<.001). With regards to ethnicity, the study population was dominated by White participants (1108/1192, 92.95%); additional ethnic groups were then consolidated into a “Nonwhite” group for subsequent analysis.

Logistic regression demonstrated that, after adjusting for age and education level, ethnicity (White vs nonwhite) was not predictive of survey mode usage (*P*=.96) (Table 4). Both age and education were significantly associated with Web-based participation versus other modes (*P*<.001 for both variables).

Each decade of increased age was associated with a 0.97-fold decrease in the odds of a patient participating on the website platform when compared to other available methods (odds ratio [OR] 0.97, *P*<.001). Each interval increase in education level saw a 1.83-fold increase in the odds of website platform usage (OR 1.83, *P*<.001). However a low *R* ^2^ value (0.06) denotes that age and education alone are poor predictors of response type, even when considering them together. Therefore, these results suggest that there are factors other than age and level of education that are likely to influence which mode people use to participate.

**Table 4:** Odds ratios from multivariate logistic regression predicting survey mode usage within each demographic variable (age, education level, and ethnicity).

Demographic Variable	Odds Ratio	Standard Error	*P*
Education^a^	1.83	0.08	< .001
Age^a^	0.97	0.01	< .001
Ethnicity,^b^ Nonwhite	1.01	0.25	.96
Constant	0.00	1.62	< .001

^a^Education and age treated as continuous/ordinal variables.

^b^White (British/French/Irish/Scottish/Welsh/[Northern, Southern, Eastern, or Western] European).

## Discussion

### Principal Results

Consistent with other single-mode survey studies, response rates were higher in the mode that was originally stated as the participant’s preferred mode [16,23]. Mail was the most preferred type of questionnaire response, and was also the most commonly used method when participants responded in a mode other than their original preference. Previous studies conducted on men and women have found that both participant attitudes toward, and familiarity with, a certain type of survey technology play an important role in their usage of that particular mode [16,24]. Therefore, it is not surprising that the mail method was commonly reverted to, especially due to the massive use of paper (mail) in our current culture. However, as Internet usage among adults continues to grow in our society, from a rate of 14% in 1995 to 87% in 2014, for example [25], we may begin to see a shift away from more traditional methods, such as mail toward Internet technologies such as Web-based questionnaires (as offered through this study), mobile applications, email, and text message. Moreover, familiarity with specific survey modes may vary across many demographic groups. For example, studies have shown that both younger participants and those with higher education are more likely to use the Internet and Web-based technologies [26-31]. This is consistent with our findings. In turn, our results did not show ethnicity to be a predictor for (or against) Web-based questionnaire usage in this study. This is consistent with a recent systematic literature review that reported research based in five multiethnic developed countries (Canada, United States, United Kingdom, New Zealand, and Australia); results found that Nonwhite populations (eg, African American, Asian, and Hispanic) who participate in surveys are as likely to participate in research as Whites [32].

For all respondents included in the Weisstock et al. study [20], a mean age of 56.6 years (SD 9.6 years) was observed, which was comparable to our subpopulation mean of 55 years (95% CI 54.4-55.5). These respondents displayed an age distribution comparable to that of women in the SMPBC and British Columbian residents at the time of the study. Our subpopulation was dominated by White participants (1108/1192, 92.95%), which included those of British / French / Irish / Scottish / Welsh / (Northern / Southern / Eastern / Western) European descent (Tables 1, 2). This is consistent with the overall study population for Weisstock et al. [20], where the most prevalent ethnicity was British/Irish/Scottish/Welsh (54% of respondents); furthermore, 49% of British Columbians self-reported ethnic backgrounds of British Isles / European / English / Scottish / Canadian / Irish / Welsh descent during the 2006 census. As with our results, age was found to influence questionnaire response mode, as younger women responded more frequently using the Web-based questionnaire, while older participants tended to respond via mail (Table 2).

As Internet technology continues to be integrated into society at various social, educational, and institutional levels, these demographic groups may not be so predictive of Web-based survey usage. While we expect the Internet to continue to become more accepted as a common means of communication, there are still many individuals, particularly those from older generations and lower education levels, who prefer mail and may be intimidated by their lack of familiarity with mobile platforms or anticipated difficulties in navigating Web-based systems [24,27]. This is especially significant in Canada, where approximately 1 in 6 Canadians are at least 65 years of age [33]. In turn, the current estimated growth rate of this population is 3.5%, approximately four times that of the total population growth rate. While many individuals within this age group may become more comfortable with technology use as they age, we must still prepare for a large proportion of this population to still prefer mail for the duration of their lifetime. Therefore, studies similar to this one will be important in future work, in order to assess how people over the age of 40, the typical age demographic for cancer diagnoses, respond to various forms of survey methods.

With regards to breast cancer, the use of alternative approaches to screening has been proposed [34]. These alternative approaches would involve making more individualized decisions based on a woman’s breast cancer risk factors and beliefs about the risks and benefits of screening mammography. This will require the collection of detailed breast cancer risk information from the eligible women, in order to estimate their risk of developing breast cancer. Web-based collection of the breast cancer risk factors may be an effective way to collect this information and, therefore, the results of this study will be of importance as personalized breast screening regimens become more main stream.

Moving forward, there is a need to protect already declining response rates [5,15,16]. The inclusion of a paper option to a Web-based survey (or vice versa) may be needed so as to prevent a premature dependency on mobile survey methods. Furthermore, it will be beneficial to develop platforms that are user-friendly and intuitive for various age groups, especially those of older generations. This is especially important for research and health service work that aims to obtain a representative sample of the population of interest. It will also be helpful to provide support through study personnel or educational materials, in order to instruct patients on how to properly use a Web-based survey system and increase their confidence with its usage [24]. Integration of such strategies will help minimize the sample bias that may typically occur should survey participation factors not be considered.

### Limitations

The predominant limitation of this study is that all participants involved were registered with the SMPBC, and were consequently adult females in the age range eligible for screening mammography (40-79 years). With the median age of Canadians being 39.9 years, we acknowledge that women within our study are generally older than the general population in Canada [35]. In turn, our population does not allow for any determination of the effect of gender on response rate and mode. Therefore, our results regarding the effects of participant preference and demographic effect on survey mode response may not be generalizable to populations that include males and females of other ages.

###  Conclusions

Our findings suggest that it is important to consider participant’s survey mode preference when designing and implementing PRO surveys. In turn, younger participants and those with higher levels of education were more likely to use the mobile platform; Web-based participation failed to vary across ethnic groups. Overall, this information will be very valuable during the planning stages of future studies. PROs are becoming increasingly used in breast cancer screening regimens and health care systems of the developed world, making it imperative that approaches to obtaining patient feedback are representative of the population being served. For this reason, clinicians and researchers must be diligent in implementing survey modes that capture data of their desired patient cohorts.

## Abbreviations

CATI: computer-assisted telephone interviewing
CI: confidence interval
HTML: HyperText Markup Language
OR: odds ratio
PRO(s): patient reported outcome(s)
SMPBC: Screening Mammography Program of British Columbia
TC: Tyrer-Cuzic
